# Education Research: Bridging the Undergraduate Neurosciences With Clinical Neurology: Neuroscience Faculty Perspectives

**DOI:** 10.1212/NE9.0000000000200005

**Published:** 2022-10-07

**Authors:** Mia Minen, Sangida Akter, Mariana Espinosa-Polanco, Raddy Ramos

**Affiliations:** From the Department of Neurology (M.M.), NYU Grossman School of Medicine; Department of Psychology (S.A., M.E.-P.), The City College of New York; and Department of Biomedical Sciences (R.R.), New York Institute of Technology, College of Osteopathic Medicine.

## Abstract

There is a significant shortage of neurologists in the United States, and this shortage is projected to worsen considerably. With the growth of undergraduate neuroscience majors, there may be opportunities to engage and motivate undergraduate students interested in the neurosciences toward clinical neurology. We surveyed undergraduate neuroscience faculty to better understand their curricular goals, existing interaction with neurologists, and their desire for additional connections with clinical neurologists and clinical neurology researchers. We invited 523 undergraduate neuroscience faculty (members of Faculty for Undergraduate Neuroscience) to complete an online survey assessing their research areas, courses taught, existing professional networks, and interest in developing connections in clinical neurology/neurology research. We had 140 of the 523 neuroscience faculty (26.8%) complete the survey. Of the 140 respondents, most respondents (93.6%, 131/140) stated their courses included a discussion about neurologic conditions, yet only 4% (6/139) stated addressing the shortage of neurologists in the country. Few reported they were able to partake in professional development opportunities for shadowing neurologists, neurosurgeons, or similar specialists prior to teaching neuroscience courses (19%, 26/140). Understanding neuroscience faculty's perspectives on how to bridge undergraduate neuroscience programs and the field of neurology is critical. This way, we can identify potential gaps and make recommendations for how to improve the neurology pipeline.

There is a shortage of neurologists in the United States, and the projected shortage is expected to significantly worsen in the years to come. In 2021, fewer than 800 graduating allopathic or osteopathic medical students in the United States applied for postgraduate year 1 (PGY1) residency positions in neurology and only 702 PGY1 residency positions in neurology were filled.^1^ By contrast, an additional 21,400 neurologists are thought to be needed by 2025, leading to a shortfall of at least 18%.^2^ Thus, although most neurology residency positions go filled each year, there are too few residency positions to fill the anticipated shortfall of clinicians.^2^ To improve the pipeline, it is necessary to understand where barriers exist in the neurology pipeline and how these might be overcome.

Recent research has shown that barriers exist in the neurology pipeline in the undergraduate/baccalaureate years. Neurology is not as visible or widely considered as a career path by undergraduate students,^3,4^ although the neuroscience major is the fifth most common undergraduate major for medical students.^5^ In fact, nearly 20% of neuroscience majors apply to medical school,^4^ yet most do not become neurologists. Being a neuroscience major in the undergraduate years increases the likelihood of pursuing a career in neurology; at the time of medical school graduation, 13.3% of those entering neurology were neuroscience majors in undergrad compared with 4.5% of those not entering neurology.^6^ Thus, despite the recent growth in neuroscience programs at the undergraduate level from fewer than 10 programs and fewer than 100 graduates in 1986^7^ to over 200 programs with over 7,000 graduates in 2018,^8^ and the connection between majoring in neuroscience and pursuing neurology, neurology is only considered as a future specialty by only 2.7% of students entering medical school.^4^

A recent study of undergraduate students interested in the neurosciences from across the United States and from 40+ undergraduate institutions reported very high levels of interest in gaining clinical neurology experience (4.66 ± 0.74), shadowing a neurologist (4.69 ± 0.76), working with neurologic patient populations (4.69 ± 0.68), and doing clinical research (4.56 ± 0.81) (all measured using Likert Scale 1–5).^9^ Two-thirds (66.7%) reported interest in conducting patient-centered clinical research. Most students (87.3%) also indicated interest in attending neurology conferences. Despite these reported interests, few students were able to have these experiences. For example, less than one-third (28.8%) had spoken with neurologists about career experience, but 95.5% of those who had the opportunity to speak with a neurologist reported that the interaction was helpful in learning about the career. Despite high interest in clinical neurology exposure, less than one-third of students had spoken with or shadowed a neurologist and only 13.6% had interacted with clinical neurology populations. Only 20.8% of students felt volunteer, and internship opportunities were sufficiently available.

With the growth in neuroscience majors at institutions across the country, an opportunity exists to develop appropriate curricula, training, mentoring, and research experiences to introduce the field of neurology and to enhance exposure to the field of neurology. Faculty who teach undergraduate neuroscience play the most important role in designing the curriculum and content disseminated in their courses. They frequently serve as faculty mentors to undergraduate premedical students, write letters of recommendation to students apply to medical school, and may serve on medical school application review committees. Gaining exposure to clinical neurology at this critical time in a person's academic path could have a significant effect on career choice. In this study, we surveyed undergraduate neuroscience faculty to better understand their curricular goals, existing interaction with neurologists, and their desire for additional connections with clinical neurologists and clinical neurology researchers.

## Methods

We conducted a cross-sectional survey of the members of the Faculty for Undergraduate Neuroscience (FUN),^10^ a professional organization dedicated to neuroscience teaching and research established in 1991.^10^ FUN focuses on promoting neuroscience undergraduate research opportunities and education.

### Survey Instrument Development

The survey was first developed and modified by a clinical neurology-researcher (M.T.M.) and undergraduate students on the team (S.A., M.E.-P., D.K., J.G., K.K.) through 2–3 collaborative sessions. Then, the survey was iteratively reviewed and revised by faculty members of FUN and the American Academy of Neurology Insights Team (full survey in eAppendix 1, links.lww.com/NXG/A543).

### Survey Distribution

The official website of FUN has a list of all faculty members (523 undergraduate neuroscience faculty). An email was sent inviting them to complete an anonymous online survey in research electronic data capture (REDCap)^11^ assessing their backgrounds (research areas and courses taught), existing professional networks, and interest in developing contacts in clinical neurology/clinical neurology research. After the initial survey invitations were emailed, reminders to complete the survey were posted on the FUN listserv and REDCap sent out autoreminder emails. Participants had 21 days to complete survey responses.

### Compensation

Participants received American Academy of Neurology (AAN) letters of recognition and access to a website with a compilation of helpful resources (e.g., upcoming conferences, internship opportunities, and journal names) for undergraduate students interested in neuroscience and clinical neurology.

### Data Analysis

The quantitative data from survey responses were analyzed in version 16.44 of Microsoft Excel, and the qualitative data was coded using grounded theory by 2 researchers on the team.^12^

### Standard Protocol Approvals, Registrations, and Patient Consents

This study was conducted with approval from the NYU Langone Institutional Review Board. The board waived the need for informed consent.

### Data Availability

Anonymized data not published within this article will be made available by request from any qualified investigator.

## Results

As presented in Table 1, between September 22, 2020, and October 9, 2020, 140 of 523 (26.8%) neuroscience faculty completed the survey. Of the 140 respondents, 58.3% (81) identified as female. Most respondents (82.6%, 114/138) were in their 30s–50s. Racially, it was a homogenous population, with White being the majority (91.4%, 117/128). There was widespread distribution of faculty across all regions of the United States, and about equal distribution between urban (35.0%, 48/137), suburban (34.3%, 47/137), and rural (30.7%, 42/137) colleges and across institutions of different sizes (52.9%, 74/140 with <5,000 students; 30%, 42/140 with 5,000–15,000 students; and 17.1%, 24/140 with >15,000 students) (see Figure, A and B for further information on institutions).

**Table 1:** Demographics of Responders and Their Respective Institutions

Demographic information	
Total responses captured, n	140
Sex (n = 139), n (%)	
Female	81 (58)
Male	57 (41)
Nonidentifying	1 (1)
Age, y (n = 138), n (%)	
20–39	34 (25)
40–59	81 (59)
60+	23 (17)
Race (n = 128), n (%)	
White	117 (91)
Black	6 (5)
Asian	4 (3)
Native Hawaiian/Pacific Islander	1 (1)
Other	2 (2)
Ethnicity (n = 138), n (%)	
Hispanic or Latino	6 (4)
Not Hispanic or Latino	132 (97)
No. of years teaching neuroscience, median (IQR)	10.6 (15.8)
Approximate no. of undergraduates in institution (n = 140), n (%)	
<5,000	74 (53)
5,000–15,000	42 (30)
>15,000	24 (17)

Abbreviation: IQR = interquartile range.

**Figure:**
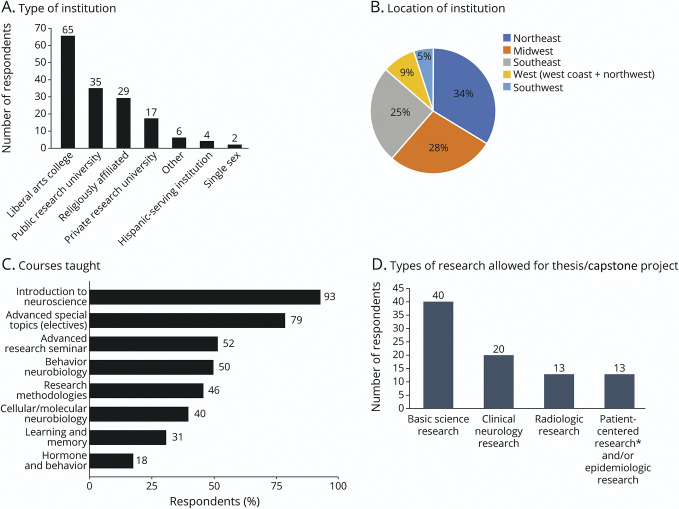
Distribution of Institutions, Faculty Courses/Research, and Undergraduate Research Requirements *Patient-centered research focuses on patients' beliefs, preferences, and needs, in contrast to clinical neurology research which focuses on the scientific study of fundamental mechanisms that underlie diseases and disorders of the brain and CNS.

### Faculty Background

Faculty respondents teach a variety of courses (Figure, C) and had been teaching any subject for a mean of 8.9 ± 10.6 years (median 10.6, interquartile range [IQR] 16) and specifically teaching in the field of neuroscience for a mean of 8.9 ± 10.6 years (median 10.6, IQR 15.8). On a 1–5-point Likert scale, faculty familiarity/comfort with clinical neurology was a median of 3 (IQR 2) (N = 139), with a median comfort level of 4 (IQR 1) (N = 140) for teaching about neurologic conditions and a median comfort level of 4 (IQR 1) (N = 55) for teaching about translational neurology research. Only 39.9% (55/138) teach students about translational neuroscience research. Neuroscience faculty conducted a variety of research, with the top 3 areas being basic neuroscience (56%, 78/140), psychology (16%, 22/140), and translational neuroscience (11%, 16/140).

### Characteristics of Neuroscience Programs in the United States

Most respondents indicated that their institution offered a neuroscience major (61%, 86/140), with some also offering a neuroscience minor (45%, 63/140). As shown in Figure, D, of those that offered students the opportunity to major in neuroscience, many require either a capstone project (41%, 35/86) or a senior thesis (21%, 18/86). For their capstone/thesis requirements, neuroscience major students were allowed to conduct research in the following areas: basic science research (100%, 40/40), clinical neurology research (50%, 20/40), radiologic research (32.5%, 13/40), and patient-centered and/or epidemiologic research (32.5%, 13/40).

### Perceptions of Neurology Research

To better understand faculty references to clinical neurology in their courses, respondents were asked to define “clinical neurology research” in the form of a free text response (Table 2). Four main themes emerged. In the first theme titled “Research of healthy humans and clinical populations,” faculty gave responses that focused on doing research of clinical populations such as “Research with humans with various neurological conditions” or “Deficits resulting from any abnormality in the brain.” There were also responses that focused on defining neurology as research of the “Brain-behavior relationship.” The second theme titled “Research on the nervous system” contained any response that mentioned the key words “nervous system.” The third theme titled “Collection of data for diagnostic or treatment application” contained responses that saw clinical neurology as a means to develop “treatments,” “diagnostic criteria,” or “medical application.” An example of an answer in this theme is “Research that has a more direct application to yielding medical treatments than research that is intended to help with understanding the healthy nervous system.” The fourth theme titled “Research on diverse subfields of neuroscience” contained responses that used neuroscience within it and referred to various subfields of it including topics related “translational neurobiology” or “epidemiology” or “neuroradiology.” Of note, 5 of the 123 respondents indicated not knowing how to define clinical neurology.

**Table 2:** Qualitative Data: Definitions of Clinical Neurology Research, Student Barriers to Finding Clinical Neurology Research Opportunities, and Advice for Undergraduate Neuroscience Students

Major themes	Subthemes
How do you define clinical neurology research?	
Research of healthy and clinical populations (121)	Research of abnormal brain functioning (64) Study of human subjects (50) Brain-behavior (7)
Research on the nervous system (NS) (24)	NS pathology (16) NS function (8)
Collection of data for diagnostic and treatment application (84)	Treatment/medical treatment (40) Diagnostic criteria/cause (17) Research (16) Mechanisms (11)
Research on diverse subfields of neuroscience (19)	Applied clinical neuroscience (5) Translational/translational neurobiology (4) Basic science (3) Basic neuroscience (2) Radiology (1) Epidemiology (1) Health neuroscience (1) Functional neuroscience (1) Clinical science (1)
Other (9)	Don't know (5) Imaging (3) Animal (1)
What have you noticed as the main barriers for your students towards finding clinical neurology research opportunities?	
Lack of exposure (52)	Lack of opportunities (32) Lack of knowledge (20)
Inadequate resources (53)	Need connections (22) Need money (13) Other college limitations (18)
Regional limitations (25)	Geographical limitations (16) Clinical setting limitations (9)
Student interest, experience, and discomfort (28)	Lack of interest (13) Lack of experience (6) Discomfort (9)
Other (15)	Logistical issues (10) No barriers (5)
If you could give a neuroscience major undergraduate student a word of advice, what would it be?	
Mindset (55)	Be creative and flexible (18) Work ethic (37)
Find as much experience as you can, through mentors, faculty, research, and internships (31)	Gain experience (22) Build connections
Take your time to find what you love and enjoy doing (45)	
Long term thinking (23)	Plan ahead (11) Things to know about the field (12)
Other (9)	Coursework suggestions (5) Career concentration suggestions (4)

### Current Opportunities

Table 3 presents opportunities for students in clinical neurology or clinical neurology research. Faculty respondents indicated that current opportunities for students in clinical neurology or clinical neurology research include shadowing a neurologist/clinical work in a neurology setting (75.8%, 75/99), research positions within an academic medical center (80.8% 80/99), and volunteering with patients with neurologic disorders (55.6%, 55/99). In addition, 24.6% (34/138) reported inviting practicing neurologists or clinical neuroscientists to visit and speak to students about their work. Of those who did not currently invite such speakers (N = 103), nearly all (99%, 102/103) were in favor of having the opportunity to do so in the future. Some faculty indicated that research opportunities were available at their institution on a student-to-student basis (91.7%, 22/24), but very few (20%, 6/30) reported that their department/program had a formal research program in place with neurologists. Most (89.3%, 25/28) faculty noted that shadowing opportunities were available to students on a case-by-case basis, but very few (24.3%, 9/37) reported that their department/program had a formal shadowing program in place with neurologists. Similarly, few faculty (28.1%, 39/139) indicated having connections with nearby neurologists for students to have a clinical neurology shadowing opportunity.

**Table 3:** Existing Opportunities for Students in Clinical Neurology or Clinical Neurology Research

	n/N (%)
Shadowing a neurologist/clinician working in a neurology setting	75/99 (75.8)
Volunteering with neurologic patients	55/99 (55.6)
Research positions with an academic medical center	80/99 (80.8)
Other	5/99 (5.1)
Have had practicing neurologists or clinical neuroscientists visit and speak to students	34/138 (24.6)
Would be open to having practicing neurologists or clinical neuroscientists visit (if there have been no visits)	102/103 (99)
Connections nearby for students to have clinical neurology shadowing opportunities	39/139 (28.1)
Department/program has a formal shadowing program in place with neurologists	9/37 (24.3)
Shadowing opportunities available on a student-to-student basis	25/28 (89.3)
Connections with neurologists nearby for students to conduct clinical neurology research projects	31/140 (22.1)
Department/program has a formal research program in place with neurologists	6/30 (20)
Research opportunities available on a student-to-student basis	22/24 (91.7)

### Barriers for Students Toward Finding Research Opportunities

Four major themes emerged from faculty's responses to the question “What have you noticed as the main barriers for your students towards finding clinical neurology research opportunities?” They were (1) lack of exposure; (2) inadequate resources; (3) regional limitations; and (4) student interest, experience, and discomfort (Table 3). The first theme “lack of exposure” was based on a lack of connections as reflected in the following responses: “connections necessary, don't know one” or “connections, we're a small school and we just don't have someone who can do the leg-work for us.” The second theme “inadequate resources” contained responses related to financial hardship/time constraints and funding limitations, which can be exemplified in this response “students need money to live on if they are to have a significant experience.” The second theme also emphasized the few opportunities available, including the competitiveness of getting opportunities as a barrier as well as the fact that researchers and physicians are often unwilling as they do not want “undergraduates in their labs” or do not “provide opportunities.” The third theme “regional limitations” included responses that mentioned geography as being a barrier like “our campus is too distant from a clinical setting.” The fourth theme “student interest, experience, and discomfort” focused on limitations originating from the students, such as a lack of interest reflected in the responses “more interested in basic research or other clinical research” or simply lack “desire.” This section also emphasizes networking and students' “discomfort with cold calling” clinicians as well as their possible insufficient academic experience being a limitation. Of note, faculty noted other additional barriers including limitations in finding opportunities due to coronavirus disease 2019 (COVID-19) and other logistical issues such as “paperwork and campus bureaucracy.” A few respondents reported no barriers.

### Advice Faculty Would Provide to Undergraduate Neuroscience Majors

Four major themes emerged from faculty's responses to the question, “If you could give a neuroscience major undergraduate student a word of advice, what would it be?” The themes were (1) mindset; (2) find as much experience as you can, through mentors, faculty, research, and internships; (3) take your time to find what you love and enjoy doing; and (4) long-term thinking (Table 2).

### Setbacks

Neuroscience faculty indicated the following as challenges students in the neuroscience major face: other required major courses too difficult (45%, 63/140), other education/life challenges not specific to the neurology major (40.7%, 57/140), required neuroscience courses are too difficult (30%, 42/140), not wanting to attend a graduate program to find a career in neuroscience (25%, 35/140), challenging to envision career options with a neuroscience major (17.9%, 25/140), and others (12.1%, 17/140).

### Neurology Exposure

All of the faculty respondents reported speaking with students about neuroscience careers, with about half (51%; 72/140) speaking frequently about this topic. Some faculty reported teaching students about translational neuroscience (39.9%, 55/138). Most (93.6%; 131/140) stated their courses included a discussion about neurologic conditions yet, only 4% (6/139) reported discussion of the neurologist shortage in the country.

### Suggestions for Improving Neuroscience Programs

The top 3 suggestions for incorporating more neurology into existing neuroscience programs were (1) funding for students to take part in neurology research experiences (81.3%, 104/128), (2) connecting with local neurologists (78.1%, 100/128), and (3) funding for sending students to neurology conferences (62.5%, 80/128).

## Discussion

This study gains insight from undergraduate neuroscience faculty who teach students about the brain and who may be the first to expose these students to neurology. Our results show that despite increases in the number of institutions offering an undergraduate neuroscience major or minor in recent years, there are currently several key barriers limiting the ability to connect the undergraduate neurosciences to clinical neurology, including faculty being neutral (not comfortable or very comfortable) regarding their familiarity/comfort with clinical neurology, lack of curricular exposure to clinical neurology, and limited resources to share with students. Undergraduate neuroscience programs could prioritize discussion of neurology/clinical neurology clerkships and electives within medical school, along with initiatives that further connect the clinical neurology and neuroscience education communities. This will likely result in an increased awareness and interest among this student population and take advantage of a time when students can make academic decisions that lead to a future career in neurology.

Neuroscience faculty do not feel comfortable with clinical neurology concepts. Thus, initiatives that grow the clinical knowledge base of neuroscience faculty may translate into greater comfort and familiarity as well as inclusion of this content into the classroom. For example, the AAN might develop an online instructional material for faculty which could then be used in their courses. Personal subscriptions or greater access to journals such as *Neurology*® would expose neuroscience faculty to up-to-date neurology research and therapeutic approaches in the field that can also augment course content. Membership to the AAN and opportunities to attend the annual AAN meeting are also strategies to increase exposure of clinical neurology among neuroscience faculty. Subspecialty societies such as the American Neuropsychiatric Association (ANPA) may also be a natural fit for exposing neuroscience faculty to clinical neurology (and clinical psychiatry). Furthermore, content from ANPA's journal titled “*Journal of Neuropsychiatry and the Clinical Neurosciences*” may be adapted for undergraduate neuroscience faculty for continuing education. Other journals including *Frontiers in Neurology*, *JAMA Neurology*, *Lancet Neurology* and *Journal for Neurology*, and *Neurosurgery and Psychiatry* might also help develop content for the faculty. Similarly, neuroscience societies such as the Society for the Neurosciences might have more continuing education and professional development opportunities with neurologists so that faculty can interact with and learn from them.

A consequence of the aforementioned barrier, the lack of faculty knowledge base about clinical neurology, is that a valuable clinical neurology material may not be incorporated by these educators into their course materials. Our findings are also supported by prior findings that unfortunately, few undergraduate neuroscience majors are exposed to clinical neurology and clinical neurology research opportunities because the curricular focus is most heavily on the basic science.^13^ The undergraduate years are a period when students often want to learn about careers applicable to their studies. Undergraduate students have expressed increased awareness of issues in medicine. Learning about the clinical application of the undergraduate work can have a profound effect on their career choice.^14^ Our results indicate that neuroscience students could be made more aware of the current shortage of neurologists in the United States and that a career in neurology could be a good fit for students interested in the brain and brain research. Currently, only 4% (6/139) of faculty initiate discussions about the present-day shortage of neurologists in the United States, and only 39.9% (55/138) teach students about translational neuroscience research. Discussion of both of these topics is key to greater understanding among neuroscience majors of career opportunities that exist for them. In addition to a general deficit in the number of neurologists in the United States today, there exists an even greater demand for underrepresented neurologists and neurology researchers.^15^ Our survey revealed that only about half (51%) of faculty members speak frequently about possible career options in the field of neurology. Thus, undergraduate neuroscience majors represent a student population for targeted outreach prior to medical school.

To increase representation and engage with diverse groups of neuroscience students, there needs to be strategic outreach, an increase in the availability of campus or classroom discussions about the present-day scarcity of neurologists, and more frequent discussions about the clinical neurology careers available to neuroscience majors. Developing courses that include neurologists as guest speakers, opportunities for students to meet people with neurologic conditions, and opportunities to discuss neurology cases might be ideal.

Currently, only 50% of faculty (20/40) reported that students have the option to pursue clinical neurology research when a capstone/thesis requirement is in place for the neuroscience major. Yet, 100% (40/40) of faculty reported that students are allowed to pursue basic science research for their capstone/thesis requirements. The reasons for this distinction and the logistical or departmental impediments students face in pursuing clinical neurology research for their culminating undergraduate research projects are important areas for further research. An initial prediction as to why colleges may discourage or prevent students from pursuing clinical neurology research projects, based on faculty's perceived barriers for students, may be the lack of practicing neurologists as faculty mentors, inability to access patient populations or inadequate funding. Establishing connections between practitioners and institutions with neuroscience programs is a direct way to open clinical research opportunities for students.

It is important that faculty cited multiple perceived barriers for undergraduate students pursuing the neuroscience major. The lack of connections with neurologists in the area may prevent the college from providing support for students who want to pursue research or shadowing opportunities. Most of the faculty participants (78.1%, 100/128) stated that a way of incorporating neurology in already existing neuroscience programs was through creating more connections with local neurologists. Only 28.1% (39/139) indicated having connections with nearby neurologists for students to have a clinical neurology shadowing opportunity. During the COVID-19 pandemic, virtual shadowing programs, virtual classrooms, and free virtual grand rounds emerged. The Duke Teleneurology Shadowing Experience^16^ and the Lenox Hill BRAINterns Program sponsored by the Department of Neurosurgery^17^ are just 2 examples of virtual shadowing opportunities for undergraduate students. Even if there are practical considerations such as confidentiality and inadequate expertise at the undergraduate level that may prevent students from taking on significant roles in patient-centered research, students should still be given opportunities to learn about the kind of clinical research occurring in the field and encouraged to develop specific avenues of interest.

Similarly, more neuroscience faculty need opportunities to supplement their classroom education with guest clinical neurologists who can provide an overview of the latest research and clinical interventions in the field. We found that while few faculty (24.6%, 34/138) currently invite clinical neurologists to speak to their students, 99% of faculty are open to doing so. These data suggest that practical barriers such as funding, lack of contacts, or geographical limitations may impede faculty from hosting clinical neurology guest speakers, the same barriers mentioned that impede students from finding clinical neurology opportunities. An important challenge for the clinical neurology community is to help establish these connections with neuroscience faculty to facilitate opportunities for clinicians to meet with students in the classroom. Guest speakers connecting remotely with students has been a recent solution to this problem and can be seen in programs such as the George Washington University Epilepsy Center Grand Rounds Series,^18^ or with New York University's course, “Grand Rounds Seminar in Child and Adolescent Mental Health Studies” which was completely remote in 2021.

An additional important limitation reported by faculty members was the lack of a formal program in place for students looking to pursue research opportunities in neurology. Multiple studies have shown that direct hands-on experience is an important factor in career choice, and one of the most important factors in graduate school admission is students' research experience.^19^ In fact, a 2019 report generated by the Association of American Medical Colleges, after surveying medical school admissions faculty on the factors that are of importance when offering interviews/acceptance offers, found that on a scale of 1–4, research experience was rated as ≥2.5 and <3.0.^20^ While opportunities for involvement in basic neuroscience research are abundant through faculty laboratories on campus or as part of thesis/capstone requirements, there are significantly fewer opportunities for engaging in clinical neurology research or patient-centered research. This may be due to an inability to access formal/informal clinical neurology research or shadowing opportunities because of the geographical location. For example, not all colleges have a medical school or large enough medical center nearby with practicing neurologists. Similarly, some campuses lack professors who are practicing clinical neurologists or research laboratories engaged in clinical neurology research. In addition, when clinical neurology research opportunities are present on campuses, they are reportedly highly competitive, and the availability of undergraduate positions is far less than the demand from students. In other cases, undergraduate students are not desired in clinical neurology research laboratories and time is spent engaging graduate students in faculty research. With the idea of creating more formal programs, there could be opportunities that are performed online or summer opportunities with possible housing or stipends for students to experience working with neurologists in a hospital or research setting.

Another major barrier perceived by faculty members as impeding students from pursuing neurology specific opportunities is the lack of funding. Lack of funding can be seen in the need for resources for travel to take part in programs, a lack of time due to working for an income, which comes hand in hand with the inability to take part in unpaid opportunities which may sometimes be the only available options. In fact, 81.3% (104/128) of faculty believed funding for neurology research opportunities is important to allow students to partake in research experiences. Students oftentimes must choose paid opportunities to support their undergraduate education. In addition, 62.5% (80/128) stated that funding to attend neurology conferences would be helpful. Student funding to attending conferences might provide opportunities for networking, overcome geographic barriers, increase exposure to clinical research, and clinician scientists and help students put their research into a larger context. These findings highlight the need for faculty, department heads, and funding bodies in the fields of neuroscience and clinical neurology to devise more formalized, paid opportunities for neuroscience students to participate in and engage with clinical neurology research and experiences.

Based on this study, future steps to increase students' interest and likelihood of pursuing a career in neurology should include creating formal undergraduate programs that provide students with funding, research, and networking opportunities with neurologists in their area. Future work might build on the recent publication which discussed how various clinician neurologists and clinician neurology researchers have integrated undergraduate students into their work. Only 20% (6/30) of faculty who reported having research programs in place for students indicated having a program specifically for neurology, and many had no programs in place at all. Funding, a shortage of networking and clinical and research opportunities in neurology, and a lack of formalized neurology programs for students are the 3 most important barriers; addressing this may go a long way to reducing the shortage of future neurologists in the country. Future work might also compare the experiences of undergraduate neuroscience faculty and students at institutions with and without an affiliated medical school to determine whether they might have different abilities and needs in establishing the clinical and research opportunities in neurology. In addition, it may be beneficial to have pipeline initiatives that help faculty who mentor premedical students by providing resources on mentorship on how to get into medical school, how to write letters of recommendation, etc.

Although there was widespread geographic distribution of respondents' colleges, generalizability of our study may be limited by our response rate of 26%. (However, this response rate is in line with other national studies of professionals published in *Neurology*.)^21-23^ In addition, most of our respondents (65%) were from Liberal Arts colleges. Those from private research universities comprised 17% of our sample. This may present a bias in respondents in saying students need more funding and research opportunities. In addition, we do not have demographic information on the nonresponders. This may limit some of the generalizability of our conclusions.

Neuroscience faculty are interested in exposing students to the field of neurology. Despite discussion of neurologic conditions in courses, there is an absence of emphasizing the need for neurologists in the United States. Undergraduate neuroscience can likely be bridged with clinical neurology by enhancing neurology shadowing, research experiences, as well as connections made in the classroom (in-person or remote). Based on this study, we suggest the development of resources to (1) increase faculty comfort with neurology, (2) enable students to network and develop relationships with neurologists and have hands-on neurology exposure, and (3) help students find sources of funding for such programs. Resources need to be available to faculty and their respective students in different geographical locations online or by providing funding for travel. Finally, participation in these programs should count toward capstone or major requirements to promote undergraduates to pursue these opportunities.

## Appendix 1 Authors

**Table:**

Name	Location	Contribution
Mia Minen, MD, MPH	Department of Neurology, NYU Grossman School of Medicine	Drafting/revision of the manuscript for content, including medical writing for content; major role in the acquisition of data; study concept or design; analysis or interpretation of data
Sangida Akter, BA	Department of Psychology, The City College of New York	Drafting/revision of the manuscript for content, including medical writing for content; major role in the acquisition of data; study concept or design; analysis or interpretation of data
Mariana Espinosa-Polanco, BA	Department of Psychology, The City College of New York	Drafting/revision of the manuscript for content, including medical writing for content; major role in the acquisition of data; study concept or design; analysis or interpretation of data
Raddy Ramos, PhD	Department of Biomedical Sciences, New York Institute of Technology, College of Osteopathic Medicine	Drafting/revision of the manuscript for content, including medical writing for content; major role in the acquisition of data; study concept or design; analysis or interpretation of data

## Glossary

AAN: American Academy of Neurology
ANPA: American Neuropsychiatric Association
COVID-19: coronavirus disease 2019
FUN: Faculty for Undergraduate Neuroscience
IQR: interquartile range
PGY1: postgraduate year 1
REDCap: research electronic data capture
